# Bilateral Neuropathy of Primary Sensory Neurons by the Chronic Compression of Multiple Unilateral DRGs

**DOI:** 10.1155/2016/2130901

**Published:** 2015-12-27

**Authors:** Ya-Bin Xie, Huan Zhao, Ying Wang, Kai Song, Ming Zhang, Fan-Cheng Meng, Yu-Jie Yang, Yang-Song He, Fang Kuang, Si-Wei You, Hao-Jun You, Hui Xu

**Affiliations:** ^1^Department of Neurobiology and Collaborative Innovation Center for Brain Science, School of Basic Medicine, Fourth Military Medical University, Xi'an 710032, China; ^2^Center for Neuron and Disease, Frontier Institutes of Life Science and of Science and Technology, Xi'an Jiaotong University, Xi'an 710049, China; ^3^Center for Biomedical Research on Pain (CBRP), College of Medicine, Xi'an Jiaotong University, Xi'an 710061, China

## Abstract

To mimic multilevel nerve root compression and intervertebral foramina stenosis in human, we established a new animal model of the chronic compression of unilateral multiple lumbar DRGs (mCCD) in the rat. A higher occurrence of signs of spontaneous pain behaviors, such as wet-dog shaking and spontaneous hind paw shrinking behaviors, was firstly observed from day 1 onward. In the meantime, the unilateral mCCD rat exhibited significant bilateral hind paw mechanical and cold allodynia and hyperalgesia, as well as a thermal preference to 30°C plate between 30 and 35°C. The expression of activating transcription factor 3 (ATF3) was significantly increased in the ipsilateral and contralateral all-sized DRG neurons after the mCCD. And the expression of CGRP was significantly increased in the ipsilateral and contralateral large- and medium-sized DRG neurons. ATF3 and CGRP expressions correlated to evoked pain hypersensitivities such as mechanical and cold allodynia on postoperative day 1. The results suggested that bilateral neuropathy of primary sensory neurons might contribute to bilateral hypersensitivity in the mCCD rat.

## 1. Introduction

Mounting evidence suggests a possible cause of low back pain and radicular pain is the mechanical deformation of the dorsal root ganglion (DRG) and its nerve roots, resulting from spinal stenosis, radiculopathies, and tumors [1]. The chronic compression of a single DRG (CCD) model mimics low back pain and radicular pain syndromes in the rat, for example, significant unilateral mechanical and heat hyperalgesia [2, 3]. However, whether CCD model exhibits spontaneous pain and cold allodynia remains unknown. Multilevel lumbosacral radiculopathies are more common than single level radiculopathies in clinic, and patients with back disorders typically exhibit multilevel nerve root compression and intervertebral foramina stenosis. In this study, the rat model of the chronic compression of unilateral multiple DRGs (mCCD) of lumbar levels 3–5 was modified. And spontaneous pain and evoked pain hypersensitivities were examined in the mCCD model. Numerous studies have shown that activating transcription factor 3 (ATF3) could be used as a neuronal marker of nerve injury [4, 5], whereas calcitonin gene-related peptide (CGRP) is a marker of nociceptive information transmission in the DRG and spinal cord [6]. Possible molecular mechanisms contributing to spontaneous pain and evoked hypersensitivities were investigated by examining plastic changes in the expression of ATF3 and CGRP in the bilateral DRGs in the mCCD rat.

## 2. Materials and Methods

### 2.1. Animals

Two-month-old male Sprague-Dawley rats (180–250 g) were obtained from the Fourth Military Medical University animal center and were maintained under standard laboratory conditions (12/12-hour light/dark cycle, 22 ± 2°C, food and water ad libitum). All animals were allowed to adapt to laboratory conditions for at least one week and were subjected to mechanical paw withdrawal threshold tests before surgery. Rats with von Frey filament mechanical thresholds between 8 and 15 grams of force were chosen and randomly divided into three groups (control, sham and mCCD groups). All procedures were in strict accordance with the guidelines established by the Fourth Military Medical University Animal Care Committee. All efforts were made to minimize animal suffering and to reduce the number of animals used.

### 2.2. Surgical Procedures

**Figure 1:**
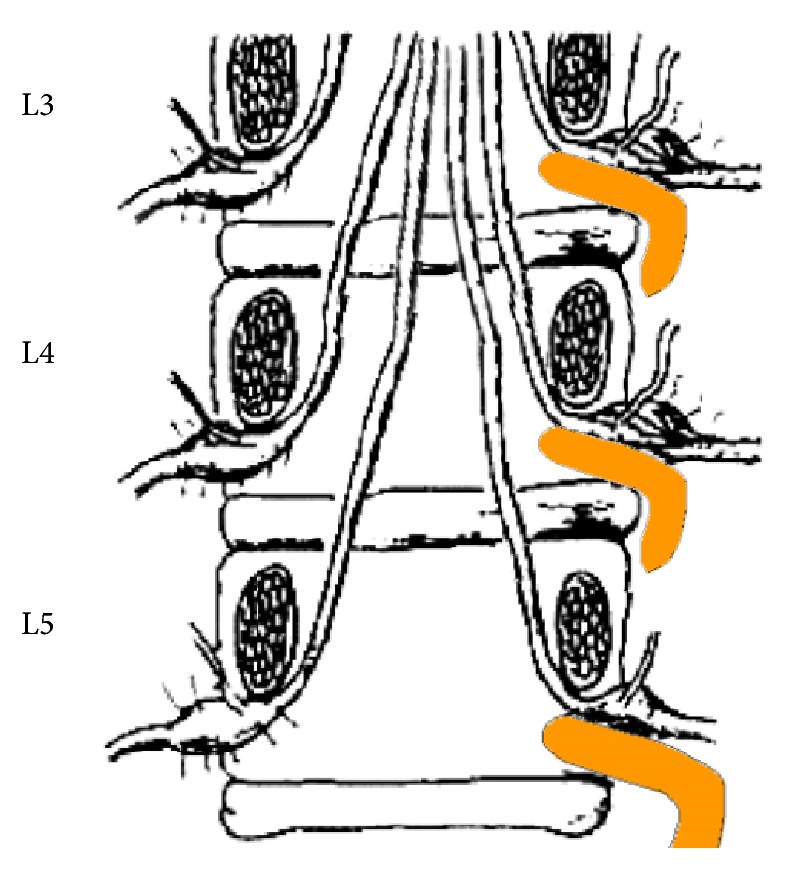
Schematic diagram of the compression of the DRG in the mCCD model.

Rats were deeply anesthetized with an intraperitoneal (i.p.) injection of sodium pentobarbital (50 mg/kg body weight). All manipulations were done on the left side of the spinal column. Special care was paid to prevent infection and to minimize the influence of inflammation. The hair of the rats' lower back was shaved and the skin was sterilized with 0.5% chlorhexidine and covered with clean gauze. Sterile surgical instruments were used. With the rats in a prone position, an incision was made along the midline of the back at the L2 and L6 spinal level. Following separation of the paraspinal muscles from the transverse process, the L3–L5 intervertebral foramina were exposed. L-shaped rods made of hollow stainless steel (4 mm in length and 0.5–0.8 mm in diameter) were carefully inserted into the L3, L4, and L5 foramina to compress the DRGs (Figure 1). In some cases, a sham surgery group of rats, the surgical procedure was identical to that for the chronic compression group, except that the stainless steel rods were not inserted into the intervertebral foramina. At the end of each study, mCCD animals were deeply anesthetized with intraperitoneal sodium pentobarbital and were dissected to verify that the compression were done at the right levels. The damage of the spinal cord at the spinal canal was examined by tracing the root to the L3–L5 DRGs after a vast dissection. Animals that had a lesion at the wrong level were excluded from the study.

### 2.3. Spontaneous Pain

Prior to testing, rats were adapted to the plastic testing chambers for at least one week (1 h per day). All tests took place between 4:00 PM and 6:00 PM, and the testing area was dimly lit to limit stress or anxiety. During behavioral assays, the experimenter was blind to the experimental treatment.

Analysis of spontaneous behavior was performed after surgery on days 1, 3, 5, 7, 14, 21, 28, and 35. All behavioral observations were performed in a low illuminated soundproof room. A sound-attenuated clear Perspex testing cage (25 × 25 × 40 cm) was fitted with a camera to record video for offline behavioral analysis. Rats were videotaped from below for 3 to 4 hours at every time point, from which 150 minutes were analyzed. Video recording started 30 minutes after the rats were placed in a cage to allow the animals to adapt to the testing conditions. A trained observer viewed the video recordings and counted the number and scored the magnitude of classified pain behaviors. The observer was trained to provide a similar rating performance (at the 95% confidence limit) of each behavior. “Wet-dog shaking” (WDS) was a behavior that resembles a wet dog that is shaking to remove water from the fur [7]. These behaviors were recorded as the number of incidences during 10-minute observation windows and summed to provide a total number of observed wet-dog shaking behaviors. Spontaneous paw shrinking behavior was recorded as the number of incidents of shrinking bilateral toes. Behaviors were recorded as number of incidences during the 10-minute observation time by using a counter, and then the total numbers of WDS and spontaneous hind paw shrinking behaviors were calculated among 150 minutes.

### 2.4. Mechanical Paw Withdrawal Threshold

Paw withdrawal thresholds to mechanical stimulation were assessed as described [8] using von Frey filaments (Stoelting Corporation, USA). Animals were placed in plastic cages with a wire mesh floor. To test for the tactile threshold required to evoke withdrawal of the stimulated paw, von Frey filaments with different bending forces (2–15.0 g) were applied perpendicularly to the plantar part of the hind paw in an ascending order. Each filament was applied 5 times to its minimum bending force, and a paw withdrawal threshold was defined as three positive responses. To avoid potential tissue damage, the cut-off threshold was assigned as 15.0 g-force.

### 2.5. Thermal Paw Withdrawal Latency

Paw withdrawal latency (PWL) to thermal stimulation was determined with a commercially available thermal Plantar Test Meter (Stoelting Corporation, USA). Rats were placed in clear plastic chambers on the top of a glass surface. The temperature of the surface was maintained at 30°C. The stimulus current of the infrared light radiant heat stimulus was maintained at 4.9 amperes while a 24-second cut-off was used to limit possible tissue damage. The time from the start of the light beam to the lifting of the paw from the glass plate (i.e., PWL) was measured. To ensure consistency, the radiant heat stimulus was always applied to the midplantar surface of the hind paw.

### 2.6. Cold Allodynia

Cold sensitivity to innocuous cold stimulation was tested by the acetone test. In brief, rats were placed in transparent plastic cages with small holes in the bottom and habituated to the test chamber for at least 30 min before the measurements. Acetone (50 *μ*L) was gently sprayed onto the plantar surface of the hind paw using a 1 mL syringe with a blunt tip needle. A brisk foot withdrawal response after an acetone spray was considered a positive response, and the responses were graded on a four-point scale: 0, no response; 1, brisk withdrawal or flick of the paw; 2, repeated flicking of the paw; and 3, repeated flicking and licking of the paw [9]. The acetone spray was applied five times with an interval of 5 min between each application. The frequency of foot withdrawal was expressed as a percentage: (number of trials accompanied by brisk foot withdrawal) × 100/(number of total trials). Acetone response scores were the average of the graded points from the five acetone spray trials.

### 2.7. Thermal Preference Task

The thermal preference task involves two temperature-regulated aluminum plates (the same as described above) that are placed end to end and separated by a piece of Plexiglass with a hole cut in it for the rat to walk through. The entire area is enclosed in a Plexiglass box (30 cm × 13 cm × 16 cm), creating two compartments whose temperatures can be independently adjusted. Thermal preference trials were 300 s and the rat was allowed to freely move back and forth between the two compartments for the entire trial. Stimulus temperatures for thermal preference testing were set at 25 and 30°C, 30 and 35°C, and 35 and 40°C, respectively. Training for the thermal preference task occurred over a four-day period with the floor plates set at 20°C for the first four trials. This allowed the rats to acclimate to the test chamber, while exploring freely from compartment to compartment and learning that the floor on each side had a different temperature. The amount of time spent on the different temperature side was recorded, respectively. A rat was considered to have crossed over when all four feet were in the new compartment.

### 2.8. Immunocytochemical Staining and Cell Counts

After the defined survival time, rats in the control (which were not treated before the surgery of the mCCD) and mCCD groups were euthanized under deep anesthesia (sodium pentobarbital, 50 mg/kg) and perfused transcardially with 0.9% saline and 4% paraformaldehyde in 0.1 M PBS (pH 7.4). The L3–L5 DRGs were removed and postfixed in the same fixative for 2 hr and then placed in 20% sucrose until they sank. The entire DRGs were cut on a cryostat into serial coronal sections at 14 *μ*m, with the first of every six sections being mounted on poly-L-lysine-coated slides and prepared for CGRP or ATF3 immunostaining.

Sections were blocked with 3% donkey serum in 0.3% Triton X-100 for 1 hr at room temperature (RT) and then incubated with rabbit anti-CGRP (1 : 1000, Sigma, USA) for 18 hr or rabbit anti-ATF3 (1 : 100, Santa Cruz Biotechnology, USA) overnight at RT. These sections were washed and incubated with fluorescently labeled goat anti-rabbit IgG (Alexa Fluor 594, Invitrogen, Life Technologies Corp., USA) for 4 hr at RT. The specificity of immunolabeling was verified by controls in which the primary antibody was omitted. Immunocytochemical double-labeling was conducted to determine the types of the cells that express CGRP or ATF3. For double- or triple-staining, the sections were incubated at RT for 18 hr in a cocktail solution containing different combinations of the following antibodies: rabbit anti-ATF3 antibody (1 : 250; Molecular Probes Inc., USA), mouse anti-neurofilament protein NF200 antibody (marker for neurons with myelinated axons; 1 : 1000; Millipore, USA), and/or mouse anti-peripherin antibody (marker for small neurons; 1 : 800, Sigma). After multiple washes in PBS, the sections were incubated at room temperature for 4 hr with fluorescently labeled goat anti-mouse IgG (Alexa Fluor 488, FITC; 1 : 500; Molecular Probes Inc., for the NF200) and/or fluorescently labeled goat anti-rabbit IgG (Alexa Fluor 594, Texas Red; 1 : 800; Molecular Probes Inc., for the ATF3), and all the sections were finally incubated at room temperature for 15 minutes with Hoechst 33342 nucleic acid stain (1 : 1000, Sigma). Sections were mounted on gelatinized slides and coverslipped.

Every sixth section throughout the DRG was processed for immunocytochemistry. All positive cells in the DRG were counted in each section at 400x magnification, omitting cells in the outermost focal plane. The total number of positive cells per section was determined and multiplied by 6 to obtain the total number of cells per DRG. The number of Hoechst-positive cells was regarded as total DRG cells in one section. For quantification of ATF3 positive, CGRP positive, and ATF3/NF200, ATF3/peripherin, CGRP/NF200, and CGRP/peripherin double-labeled cells, immunofluorescence images were obtained under a BX51 fluorescence microscope (Olympus, Japan). The percentage of ATF3 or CGRP positive cells per section was the number of ATF3 or CGRP positive cells divided by the number of Hoechst-positive cells. The percentage of double-staining of ATF3 with NF200 or peripherin was calculated among NF200- or peripherin-positive neurons, respectively. Similarly, the percentage of double-staining of CGRP with NF200 or peripherin was calculated among NF200- or peripherin-positive neurons, respectively.

### 2.9. Statistical Analysis

**Figure 2:**
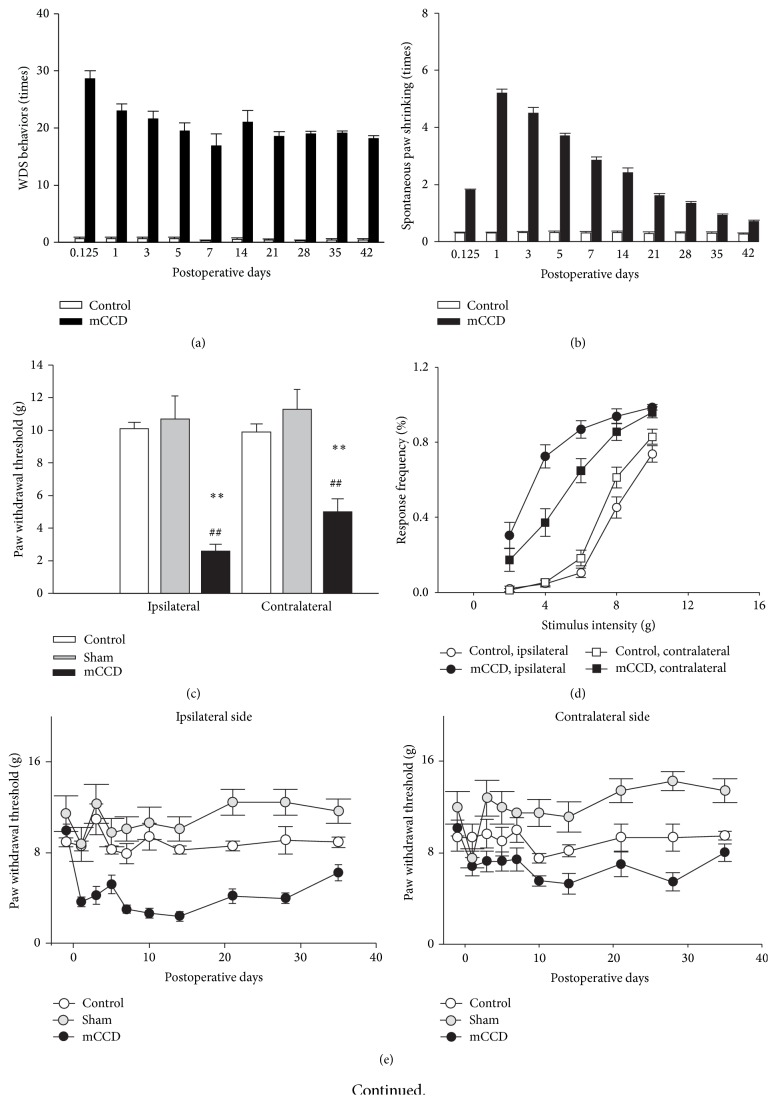
Behaviors of spontaneous pain, mechanical allodynia, and hyperalgesia recorded in mCCD rats. (a) The increase of the number of “wet-dog shaking” (WDS) behaviors at different time points after the mCCD (RM ANOVA, *P* < 0.01). (b) The increase of the number of shrinking bilateral hind paws at different time points after the mCCD (RM ANOVA, *P* < 0.01). (c) Hind paw withdrawal thresholds were markedly decreased bilaterally after the chronic compression (*n* = 29) compared to those of control (*n* = 35) and sham groups (*n* = 6) (on 10th postoperative day, one-way ANOVA followed by Bonferroni tests, ^*∗∗*^ *P*s < 0.01 compared to those of control group, ^##^ *P*s < 0.01 compared to those of sham group). (d) Stimulus response curves of ipsilateral and contralateral hind paws to von Frey filaments. Following the chronic compression of multiple DRGs (*n* = 10), the stimulus response curves of both hind paws were markedly shifted to the left compared to those of the control group (*n* = 6) (general line model, all *P*s < 0.05), and there was an obvious significant increase in the response frequency to 2.0 and 6.0 g von Frey filaments (nonnoxious mechanical stimuli) of both the ipsilateral and contralateral sides (Bonferroni test, *P*s < 0.01). There was also a significant increase in the response to 8.0 and 10.0 g von Frey filaments (which were noxious mechanical stimuli) bilaterally (Bonferroni test, *P*s < 0.01). (e) The time course of ipsilateral and contralateral hind paw mechanical withdrawal thresholds after chronic compression of multiple DRGs (mCCD group: *n* = 29; control group: *n* = 35; sham group: *n* = 6). The ipsilateral hind paw withdrawal thresholds were decreased significantly below baseline on the 1st postoperative day and remained low throughout the entire testing period (RM ANOVA, *P*s < 0.01). Contralateral mechanical thresholds were similarly decreased to a lesser extent on the 1st postoperative day, reached their lowest peak during the 10th to 14th postoperative day (RM ANOVA, *P*s < 0.05), and recovered to basal levels thereafter. (f) Bilateral mechanical hypersensitization occurred in bilateral forepaws in the mCCD rat (mCCD group: *n* = 4; control group: *n* = 4; RM ANOVA, *P*s < 0.05).

All results were expressed as mean ± SEM. Statistical analyses of pain behaviors were performed with a repeated measures analysis of variance (RM ANOVA). The difference of pain behaviors between bilateral sides was analyzed with one-way ANOVA followed by Bonferroni multiple comparison* post hoc* tests. General linear model was used on data in Figure 2(d). Immunocytochemical data in Figures 5(b), 6(b), 7(b), and 8(c)–8(e) were analyzed with general linear model followed by Bonferroni multiple comparison* post hoc* test. Linear regression analysis was performed on data in the Supplementary Figures in Supplementary Material available online at http://dx.doi.org/10.1155/2016/2130901. Data management and statistical analyses were performed using SPSS (v14.0). Statistical significance was set at *P* < 0.05.

## 3. Results

### 3.1. Spontaneous Pains

During the period of behavioral testing of up to five weeks, a total of 95 rats with the chronic compression of multiple DRGs, 6 sham rats, and 80 control naive rats without any surgery appeared in good health. Rats were awake within several minutes after anesthesia was terminated and responsive an hour later. They gained weight during the test period and were well groomed and exhibited no self-inflicted wounds. Three hours after the compression of multiple DRGs, rats exhibited a marked guarding behavior of leaning to the healthy side to minimize weight bearing of the injured hind paw. This phenomenon lasted throughout the observation period (the longest period was 42 days) of the present study. Videotape analysis confirmed that the incidence of wet-dog shaking (WDS) was significantly increased on postoperative days 1, 3, 5, 7, 14, 21, 28, 35, and 42, respectively, compared to those of normal control rats (mCCD group: *n* = 8; control group: *n* = 8; Figure 2(a), RM ANOVA, *P* < 0.01). Moreover, spontaneous lifting of bilateral feet was increased for mCCD rats at all time points and statistically significantly different to those of normal rats at day 1 to day 42 compared to those of normal control rats (mCCD group: *n* = 8; control group: *n* = 8; Figure 2(b), RM ANOVA, *P* < 0.01). No rats in any groups exhibited the autotomy that could occur after nerve transection.

### 3.2. Bilateral Mechanical Allodynia and Hyperalgesia

Withdrawal thresholds to mechanical stimulation of the plantar surface of the hind paws were examined in mCCD rats (*n* = 29) on days 1, 3, 5, 7, 10, 14, 21, 28, and 35, postoperatively, and in normal control animals (*n* = 35). This increase in mechanical sensitivity was reflected in the large bilateral decrease in the hind paw withdrawal thresholds (defined as the minimum bending force required to elicit 50% response incidence) after the mCCD (Figure 2(c), ipsilateral: 4.0 ± 0.4 g; contralateral: 6.0 ± 0.5 g, day 10 compared to those of the control and sham groups) (Figure 2(c), control group: ipsilateral side, one-way ANOVA followed by Bonferroni test, *P* < 0.01, contralateral side, one-way ANOVA followed by Bonferroni test, *P* < 0.01; sham group: ipsilateral side, one-way ANOVA followed by Bonferroni test, *P* < 0.01, contralateral side, one-way ANOVA followed by Bonferroni test, *P* < 0.01). In control animals (*n* = 35), hind paw mechanical withdrawal thresholds were 9.7 ± 0.4 g (left side) and 9.9 ± 0.5 g (right side), and there was no significant difference between sides (Figure 2(c), one-way ANOVA followed by Bonferroni test, *P* > 0.05). Similarly, hind paw mechanical withdrawal thresholds were 10.7 ± 1.4 g (left side) and 11.3 ± 1.2 g (right side), and there was no significant difference between sides (Figure 2(c), one-way ANOVA followed by Bonferroni test, *P* > 0.05). The mean incidence of left and right hind paw withdrawal responses to von Frey filament stimulation increased monotonically with increases in bending force within the range of 2.0 to 10.0 g (Figure 2(d)). There were statistically significant interactions between every two factors of sides, groups, and intensities (general line model, all *P*s < 0.05). There was no significant difference in the mechanical sensitivity between hind paws preoperatively (Figure 2(d), Bonferroni test, *P* > 0.05). Following the chronic compression of multiple DRGs, the stimulus response curves of both the left (ipsilateral) and right (contralateral) hind paws were markedly shifted to the left compared to those of the control group (Figure 2(d), day 10, Bonferroni test, *P*s < 0.05). The increase in mechanical sensitivity was significant for 2.0 and 6.0 g von Frey filaments (nonnoxious mechanical stimuli) bilaterally (Figure 2(d), Bonferroni test, *P*s < 0.01). Moreover, the increase in the response frequency was significant for 8.0 and 10.0 g von Frey filaments (which were noxious mechanical stimuli) as well, for both the ipsilateral and contralateral sides (Figure 2(d), Bonferroni test, *P*s < 0.01). The time course of mechanical withdrawal thresholds of the ipsilateral and contralateral hind paws following the mCCD was shown in Figure 2(e). Following the mCCD, the withdrawal thresholds of the ipsilateral hind paw decreased significantly on the 1st postoperative day and remained below baseline throughout the entire testing period (Figure 2(e), RM ANOVA, *P* < 0.01). The contralateral mechanical thresholds decreased but to a lesser extent starting on the 1st postoperative day. Contralateral mechanical sensitivity peaked 10 to 14 days postoperatively and subsequently recovered to the basal preoperative levels by day 35 (Figure 2(e), RM ANOVA, *P* < 0.05). There was also significant difference between ipsilateral and contralateral mechanical sensitivity in the mCCD (one-way ANOVA followed by Bonferroni test, *P* < 0.01). In contrast, hind paw mechanical withdrawal thresholds did not significantly change in control rats throughout the testing period (Figure 2(e), RM ANOVA, *P* > 0.05). Similarly, the trend of bilateral mechanical hypersensitization occurred in bilateral forepaws in the mCCD rat (mCCD group: *n* = 4; control group: *n* = 4; Figure 2(f), RM ANOVA, *P* < 0.05).

### 3.3. Bilateral Thermal Hyperalgesia

**Figure 3:**
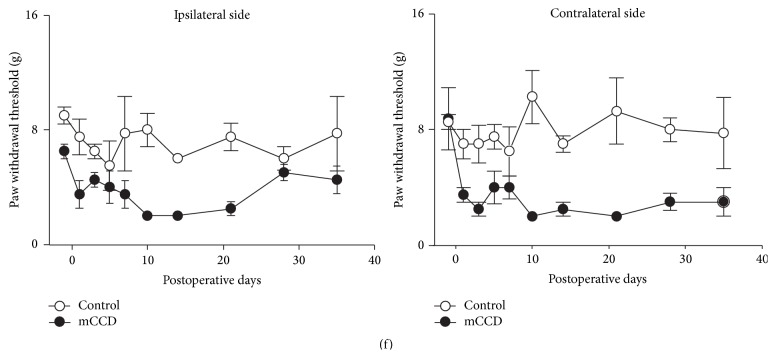
Thermal hyperalgesia and cold allodynic behaviors in the mCCD rat. (a) The time course of ipsilateral and contralateral hind paw withdrawal latencies to radiant thermal stimulation in mCCD (*n* = 8) and control rats (*n* = 8). Following the chronic compression of multiple DRGs, ipsilateral and contralateral hind paw withdrawal latencies were transiently decreased from the 1st postoperative day to postoperative day 21 compared to those of control groups (RM ANOVA, ipsilateral side: *P* < 0.05; contralateral side: *P* < 0.05). After 21 days, hind paw withdrawal latencies recovered to the baseline preoperative levels. (b) The time course of ipsilateral and contralateral forepaw withdrawal latencies to radiant thermal stimulation in mCCD (*n* = 4) and control rats (*n* = 4). No significant changes in either ipsilateral or contralateral forepaw withdrawal latencies in the mCCD rat (RM ANOVA, *P*s > 0.05). (c) Response scores of the ipsilateral and contralateral hind paws to acetone in control (*n* = 23) and mCCD (*n* = 16) rats. Both ipsilateral and contralateral response scores to acetone increased after the chronic compression of multiple DRGs compared with the control group (one-way ANOVA followed by Bonferroni test, ipsilateral: *P* < 0.001; contralateral: *P* < 0.01). (d) Response frequencies of the ipsilateral and contralateral hind paws to acetone in control and mCCD rats. Cold allodynic response frequencies of both ipsilateral and contralateral hind paws were significantly increased in chronically compressed rats compared to that of the control group (one-way ANOVA followed by Bonferroni test, ipsilateral: *P* < 0.01; contralateral: *P* < 0.01).

Hind paw withdrawal latencies (PWLs) to radiant thermal stimulation were examined in normal and mCCD rats (on presurgical and testing/postoperative days 1, 3, 5, 7, 10, 14, and 21, resp.) and analyzed separately for each hind paw (Figure 3(a)). Before surgery, the mean latencies were 12.0 ± 0.4 s (left side, *n* = 14) and 11.9 ± 0.5 s (right side, *n* = 14), and no significant difference was observed between the 2 sides (one-way ANOVA followed by Bonferroni test, *P* > 0.05). Following the chronic compression of multiple DRGs, the ipsilateral hind paw withdrawal latencies were transiently decreased from postoperative day 1 to day 14 (RM ANOVA, *P* < 0.001) that subsequently recovered to the basal preoperative levels on postoperative day 21 compared to that of control rats. The contralateral hind paw thermal latencies exhibited a delayed decrease from postoperative day 3 and returned to the baseline on postoperative day 21. There was also a significant difference between ipsilateral and contralateral thermal sensitivity in the mCCD (one-way ANOVA followed by Bonferroni test, *P* < 0.01). In contrast, hind paw withdrawal latencies did not significantly change in control rats throughout the testing period (*n* = 7, RM ANOVAs, *P* > 0.05) and there were no significant differences between thermal withdrawal latencies of left and right hind paws in control rats (one-way ANOVA followed by Bonferroni test, *P* > 0.05). However, there were no significant differences in either ipsilateral or contralateral thermal sensitivity of forepaws in the mCCD on postoperative days 8 and 15 compared to those of control rats (mCCD group: *n* = 4; control group: *n* = 4; Figure 3(b), RM ANOVAs, *P*s > 0.05).

### 3.4. Bilateral Cold Allodynia

Cold sensitivities of the ipsilateral and contralateral hind paws to acetone were tested prior to surgery and on day 10 after the mCCD. Before surgery, cold allodynic response scores of the hind paws to acetone were 0.8 ± 0.1 (left, *n* = 16) and 0.9 ± 0.1 (right, *n* = 16), and no significant difference existed between the left and right sides (Figure 3(c), one-way ANOVA followed by Bonferroni test, *P* > 0.05). Similarly, there was no significant difference in the cold allodynia response frequencies between the two hind paws (Figure 3(d), 0.5 ± 0.1 (left, *n* = 16) and 0.6 ± 0.1 (right, *n* = 16), one-way ANOVA followed by Bonferroni test, *P* > 0.05). After the chronic compression of multiple DRGs, both ipsilateral and contralateral response scores to acetone were markedly increased when compared with the control group (Figure 3(c), ipsilateral: 2.1 ± 0.1, *n* = 16, one-way ANOVA followed by Bonferroni test, *P* < 0.01; contralateral: 1.6 ± 0.2, *n* = 16, one-way ANOVA followed by Bonferroni test, *P* < 0.01). There was no significant difference between ipsilateral and contralateral response scores to acetone in mCCD rats. Similarly, allodynic response frequencies of both ipsilateral and contralateral hind paws were significantly increased in chronically compressed rats (Figure 3(d), ipsilateral: 0.9 ± 0.1, *n* = 16, one-way ANOVA followed by Bonferroni test, *P* < 0.01; contralateral: 0.8 ± 0.1, *n* = 16, one-way ANOVA followed by Bonferroni test, *P* < 0.01, compared to that of control group). There was no significant difference between ipsilateral and contralateral cold response scores and frequencies in the mCCD (one-way ANOVA followed by Bonferroni test, *P*s > 0.05).

### 3.5. Thermal Preference to a Low-Temperature Plate between 30 and 35°Cs

**Figure 4:**
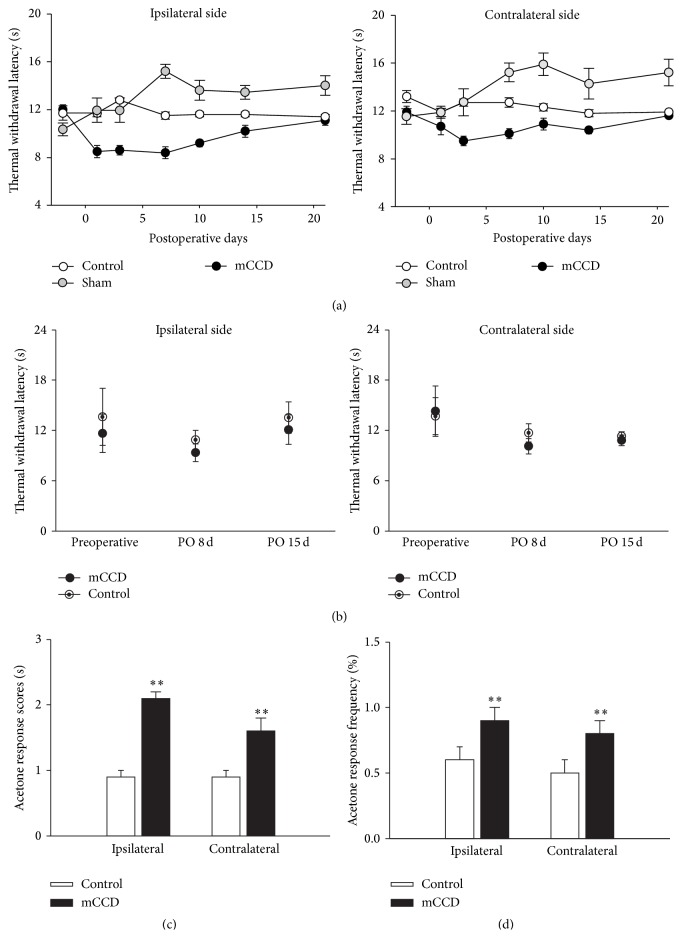
Decreased thermal preference to 30°C plate during the range of 30 and 35°C in mCCD rats. (a) Similar thermal preference of 30°C between control and mCCD rats to the temperature of 25 and 30°C (two-way ANOVA, *P* < 0.01). (b) Decreased thermal preference to 30°C of thermal preference in mCCD to the temperature of 30 and 35°C (two-way ANOVA, *P* < 0.01). (c) Similar thermal preference of 35°C between control and mCCD rats to the temperature of 35 and 40°C (two-way ANOVA, *P* < 0.01).

On 25°C and 30°C plates, mCCD (*n* = 3) and control (*n* = 4) rats preferred staying on 30°C plates (Figure 4(a), repeated measures analysis of variance, *P* < 0.01). MCCD rats preferred spending significantly more time on the less hot (30°C) side of plate than control naive rats on 35°C plates (Figure 4(b), repeated measures analysis of variance, *P* < 0.01), whereas mCCD and control rats responded the same and preferred staying on 35°C plate on either 35°C or 40°C plates (Figure 4(c), repeated measures analysis of variance, *P* < 0.01). All these trends were not changed during the period of postoperative days 1 to 42.

### 3.6. The ATF3 Expression in Ipsilateral and Contralateral DRGs

**Figure 5:**
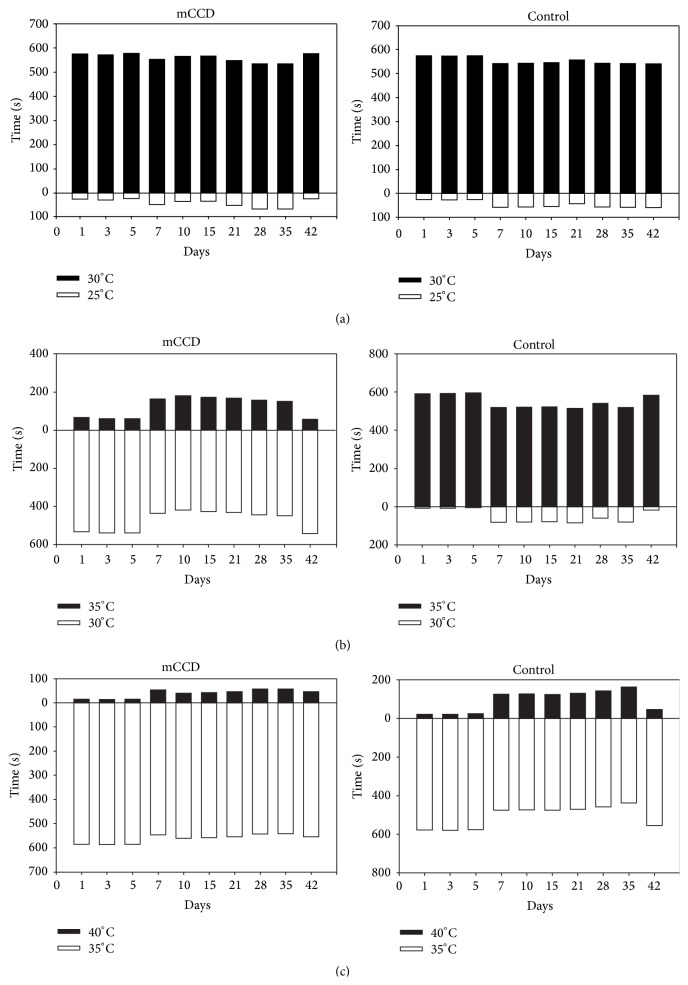
ATF3 expression was markedly increased in both ipsilateral and contralateral DRG neurons following the mCCD. (a) ATF3 expression in ipsilateral and contralateral DRGs in control and mCCD rats on 1st, 7th, 10th, 14th, and 21st postoperative days (inset: higher magnification, scale bar = 20 *μ*m). (b) Percentage of ATF3 positive cells in ipsilateral and contralateral DRGs following the multiple DRG compression. Scale bar = 50 *μ*m. Bonferroni test, *P*s < 0.001, compared to the bilateral DRG neurons of control rats.

**Figure 6:**
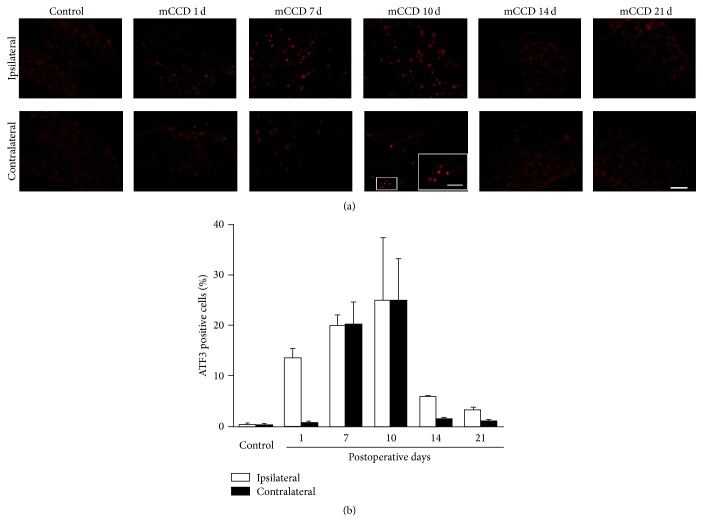
Increased ATF3 expressions in ipsilateral and contralateral large- and medium-sized DRG neurons following the mCCD. (a) ATF3 expression in ipsilateral large- and medium-sized DRG neurons of control rats. (b) ATF3 expression in ipsilateral large- and medium-sized DRG neurons at day 10 after mCCD surgery. C, ATF3 expression in contralateral large- and medium-sized DRG neurons at day 10 after mCCD surgery (inset: higher magnification, scale bar = 20 *μ*m). D, Percentage of ATF3 positive cells in ipsilateral and contralateral large- and medium-sized DRG neurons in control and on the 1st, 7th, 10th, 14th, and 21st postoperative days. Scale bar = 50 *μ*m (a–C). Bonferroni test, *P*s < 0.001, compared to the bilateral DRG neurons of control rats.

**Figure 7:**
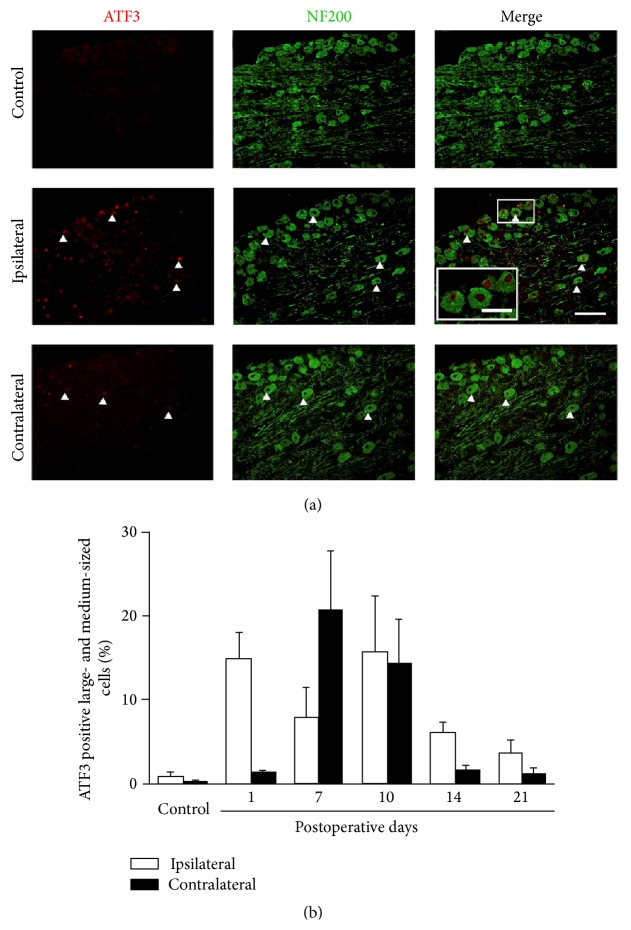
Increased ATF3 expressions in ipsilateral and contralateral small-sized DRG neurons following the mCCD. (a) ATF3 expression in ipsilateral small-sized DRG neurons of control rats. (b) ATF3 expression in ipsilateral small-sized DRG neurons at day 10 after mCCD surgery. C, ATF3 expression in contralateral small-sized DRG neurons at day 10 after mCCD surgery (inset: higher magnification, scale bar = 20 *μ*m). D, The percentage of ATF3 positive cells in ipsilateral and contralateral small-sized DRG neurons in control and on the 1st, 7th, 10th, 14th, and 21st postoperative days. Scale bar = 50 *μ*m (a–C). Bonferroni test, *P* < 0.001, compared to the bilateral DRG neurons of control rats.

**Figure 8:**
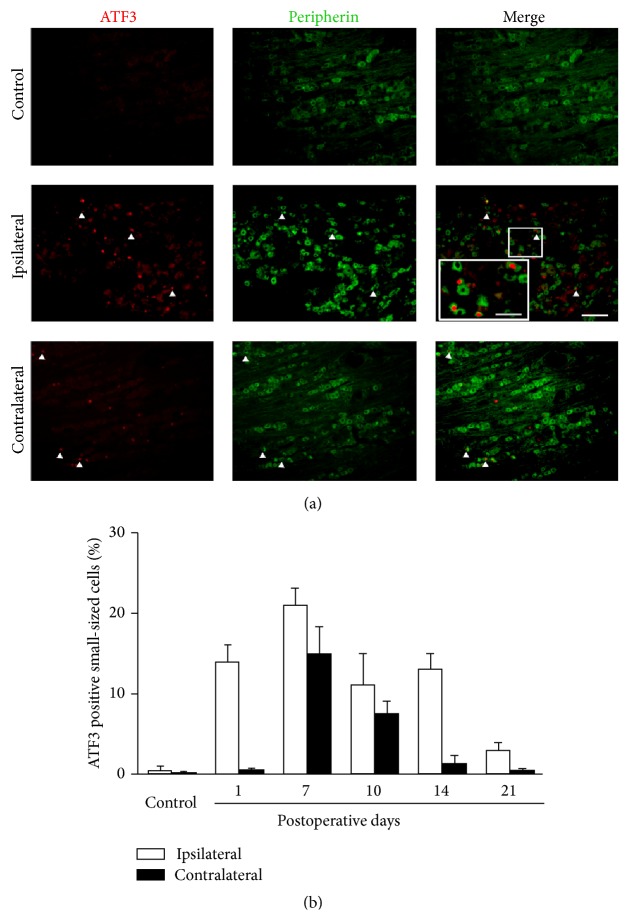
CGRP expression was significantly increased in large- and medium-sized neurons but not in small-sized neurons in ipsilateral and contralateral DRGs following the mCCD. (a) CGRP expression in ipsilateral large- and medium-sized DRG neurons in control and mCCD rats (on 10th postoperative day, inset: higher magnification, scale bar = 20 *μ*m). (b) CGRP expression in ipsilateral small-sized DRG neurons in control and mCCD rats (on 10th postoperative day; inset: higher magnification, scale bar = 20 *μ*m). (c) Percentage of CGRP positive cells in ipsilateral and contralateral DRGs following the multiple DRG compression on the 1st, 7th, 10th, 14th, and 21st postoperative day. (d) The percentage of CGRP positive cells in ipsilateral and contralateral large- and medium-sized DRG neurons was significantly increased following the chronic compression of DRGs. (e) The percentage of CGRP positive small-sized neurons in ipsilateral and contralateral DRGs was significantly increased following the chronic compression of DRGs. Scale bar = 50 *μ*m (a-b). Bonferroni test, *P*s < 0.001, compared to the bilateral DRG neurons of control rats.

Following the mCCD, ATF3 expression was investigated in ipsilateral and contralateral DRGs postoperatively on days 1, 7, 10, 14, and 21. Before the surgery, in normal naive rats, which were not treated, ATF3 was investigated in L3–L5 DRGs at the starting day. There was hardly any expression of ATF3 in L3–L5 DRG neurons (Figure 4(a)). No significant difference in the expression of ATF3 was detected between the L3–L5 DRGs (data not shown). There were no statistically significant interactions between the factors of sides and days and the factors of sides and immunostaining markers (Figures 5–8, general line model, all *P*s > 0.05); however, there were statistically significant interactions between the factors of immunostaining markers and days (Figures 5(a) and 5(b), general line model, *P* < 0.001). Following the mCCD, the expression of ATF3 markedly increased in both ipsilateral and contralateral L3–L5 DRG neurons on postoperative days 1, 7, 10, and 14 (Figures 5(a) and 5(b), Bonferroni tests, *P*s < 0.001), and there was no statistical significance between the ATF3 expressions of the ipsilateral and contralateral DRGs (Figures 5(a) and 5(b), Bonferroni test, *P* > 0.05). For a side there was no difference in the expression of ATF3 among the L3, L4, and L5 DRGs (data not shown). A significant increase of ATF3 expression in bilateral DRG neurons gradually decreased to a low level on day 21 (Figures 5(a) and 5(b), Bonferroni tests, *P*s > 0.05). There was a trend of delayed increase in ATF3 expression of contralateral DRGs compared to that of ipsilateral DRGs.

Immunostaining with NF200, to label large- and medium-sized DRG cells, and peripherin, to identify small-sized DRG neurons, was used to further investigate which cell types were expressing ATF3 following the mCCD. In normal control rats, there was little expression of ATF3 in large- and medium-sized DRG neurons in L3–L5 DRGs and no difference was detected in the ATF3 expression between sides (Figure 6, Bonferroni test, *P* > 0.05). Following the mCCD, the expression of ATF3 was markedly increased in ipsilateral large- and medium-sized DRG neurons (day 10, shown in Figure 6(a)) and reached a peak on day 10 and gradually decreased to a stable level till day 14 (Bonferroni test, *P* < 0.001). The expression of ATF3 was significantly increased in contralateral large- and medium-sized DRG neurons on day 7, reached the peak on day 10 (Bonferroni test, *P* < 0.001), and gradually decreased to a low level on day 21 (Bonferroni test, *P* > 0.05). A trend of delayed increase in contralateral large- and medium-sized and small-sized DRG neurons occurred compared to the expression of ipsilateral DRG neurons following the chronic compression of the DRGs.

In small-sized DRG cells, the expression of ATF3 was significantly increased in ipsilateral DRG neurons on day 1, reached a peak on day 7, and gradually decreased to a stable level till day 14 (Figure 7, Bonferroni test, *P* < 0.001), which then returned to basal levels on day 21 (Figure 7, Bonferroni test, *P* > 0.05). In the contralateral small-sized DRG neurons, the expression of ATF3 was markedly increased on day 7, was decreased on day 14 (Figure 7, Bonferroni test, *P* < 0.001), and was returned to basal levels by day 14 (Figure 7, Bonferroni test, *P* > 0.05).

### 3.7. The CGRP Expression in Ipsilateral and Contralateral DRGs

Following the mCCD, the expression of CGRP, a neuropeptide related to nociceptive information transmission, was investigated in ipsilateral and contralateral DRGs on postoperative days 1, 7, 10, 14, and 21. In normal control rats, 30% of the lumbar DRG neurons expressed CGRP (Figure 8). There was no marked difference in the CGRP expression between the L3–L5 DRG neurons (data not shown). Compared with the control group, the expression of CGRP significantly was increased in both ipsilateral and contralateral DRGs after the mCCD (Figure 8(c), Bonferroni test, *P* < 0.05). An increase of CGRP expression in ipsilateral DRG neurons occurred rapidly on postoperative day 1 and remained at a high level through day 21 (Bonferroni test, *P* < 0.001). A similar increase of CGRP expression in contralateral DRG neurons occurred on postoperative day 7 and remained at a high level till day 21 (Bonferroni test, *P* < 0.001).

Following the mCCD, the expression of CGRP was markedly increased (day 10, shown in Figure 8(a)) in bilateral large- and medium-sized DRG neurons and reached a peak on day 10 and gradually decreased to a stable level till day 21 (Bonferroni test, *P* < 0.001). A trend of decrease of CGRP expression in ipsilateral small-sized DRG neurons occurred rapidly on postoperative day 1 and remained at a low level through day 21 (Bonferroni test, *P* < 0.001). A trend of a delayed increase of CGRP expression in contralateral small-sized DRG neurons occurred on postoperative day 14 and remained at a high level till day 21 (Bonferroni test, *P* < 0.001).

The extent of neuronal injury was measured by the expression of ATF3 in the DRGs in order to investigate the relationship of the expression of pain behaviors to neuronal injury after the mCCD (*n* = 5). Linear regression lines relating the extent of hypersensitive behaviors and the percentages of ATF3 positive DRG neurons were almost perfectly superimposed for the bilateral mechanical and cold allodynia (Supplementary Figures 1A and 1B, linear regression analysis, *P*s < 0.05). The results indicated a strong influence of the number of damaged sensory neurons on the bilateral allodynic behaviors on 1st postoperative day (Supplementary Figure 1, *P* < 0.05). Similarly, significant correlations between the expression of CGRP in the DRGs and evoked pain hypersensitive behaviors were observed after the chronic compression of multiple DRGs. The extents of evoked pain hypersensitive behaviors (mechanical and cold allodynia) were positively correlated with the CGRP expression in both ipsilateral and contralateral DRG neurons (*n* = 5, Supplementary Figures 2A and 2B), indicating an influence of the number of sensory neurons conducting nociceptive information on evoked pain behaviors.

## 4. Discussion

Our study showed that mCCD rats exhibited spontaneous pain, contralateral mechanical allodynia, mechanical and thermal hyperalgesia, and cold allodynia similar to the ipsilateral hypersensitivity, as well as thermal allodynia. Moreover, the expression of ATF3 and CGRP was upregulated in ipsilateral and contralateral DRGs. The extent of evoked mechanical and cold allodynia was positively correlated with the upregulated expression of ATF3, a neural injury marker, and CGRP, a marker of nociceptive information transmission, in bilateral DRG neurons, respectively.

### 4.1. Behavioral Signs of Spontaneous Pain and Bilateral Hypersensitivities

Spontaneous pain is a very serious symptom in patients with neuropathic pain. One proposed biomarker of spontaneous pain is autotomy, a behavior frequently observed in rats with complete hind paw denervation or neuroma models [10, 11], but this rarely occurs in models of partial paw denervation, such as SNL and partially sciatic ligation [12, 13]. In the present study, there were marked and rapid increases of the numbers of wet-dog shaking and spontaneous hind paw shrinking behaviors following the mCCD, indicating that mCCD rats developed a rapid and lasting spontaneous pain in addition to the evoked pain behaviors, but without autotomy. This is the first time the spontaneous pain in the mCCD rat is reported. Our results showed that the increased change of bilateral spontaneous toe shrinking behavior lasted till postoperative day 42 and resembled the change of wet-dog shaking behavior after the mCCD. It is also suggested that bilateral spontaneous hind paw shrinking behaviors were able to be used to evaluate spontaneous pain of the rat.

It is interesting to note that the mCCD model displayed early and long-lasting contralateral mechanical allodynia and hyperalgesia in addition to ipsilateral allodynia and hyperalgesia, which differs from the CCD model of a single DRG [2, 3], and the contralateral hypersensitivity occurred earlier and to a greater extent compared to the CCD model [14]. Moreover, the contralateral mechanical allodynia occurred not only in hind paws but also in forepaws, indicating that a supraspinal mechanism might be involved in the mCCD rat. In addition, contralateral mechanical allodynia and hyperalgesia were much less strong than ipsilateral evoked pain behaviors. Contralateral mechanical allodynia has been shown in animal models of neuropathic and inflammatory pain, including unilateral spinal nerve ligated rats [13, 15] and the spared nerve injury model [16]. Similarly, significant contralateral mechanical hyperalgesia and allodynia have been also reported in the ultraviolet- (UV-) B irradiated inflammation model [17]. Previous studies provided evidence that the duration and intensity of bilateral pain are dependent on the severity of the neural injury [18–20]. Our present data showed that significant increased expression of ATF3 was observed in all-sized neurons in the ipsilateral and contralateral DRGs in the mCCD rat. Increased ATF3 expression was more delayed in contralateral DRGs on postoperative day 7 compared to that of ipsilateral DRGs. This significant increased expression of ATF3 was observed in all-sized neurons in the ipsilateral and contralateral DRGs. Furthermore, there were significant correlations between ATF3 expression and evoked pain behaviors such as mechanical and cold allodynia on postoperative day 1. Our results suggested that both ipsilateral and contralateral neuronal damage in the periphery might contribute to bilateral mechanical and cold allodynia in the mCCD rat. Those results were consistent with contralateral neuropathology of the DRG in a rat model of noncompressive disc herniation [4]. This might be a reason that marked bilateral mechanical allodynia and hyperalgesia developed due to the extensive neuronal damage of ipsilateral and contralateral DRGs after the mCCD, while there were no or mild contralateral allodynia and hyperalgesia in the CCD model because of the single DRG affected.

The mCCD rat exhibited delayed and transient contralateral thermal hyperalgesia in the hind paws on postoperative day 3 that lasted up to 14 days, inconsistent with the results described after lumbar 5 ventral root transection of the rat lasting for a long period [21]. The phenomenon did not happen in the forepaws. This result indicated that peripheral or spinal mechanisms might contribute to contralateral heat hyperalgesia. In addition, bilateral cold allodynia occurs in the mCCD rat as well. Cold allodynia is one of the most common symptoms among sympathetic-maintained pain patients. Up till now, there were few animal models that display this phenomenon. Contralateral cold allodynia was shown for the L4 and L5 SNL model, but not for the L5 SNL model [9, 22]. This suggests contralateral cold allodynia possibly correlates with the extent of peripheral neuropathy. Our results also showed mCCD rats exhibited a decreased thermal preference. Taken together, the mCCD rat exhibits many characteristics of bilateral hypersensitivity including mechanical, cold, and thermal allodynia and thermal hyperalgesia as well.

### 4.2. Peripheral Mechanism of Contralateral Hypersensitivity

Previous studies demonstrated that hyperexcitability of DRG neurons was involved in the development or maintenance of chronic neuropathic pain [23]. Plastic changes of DRG neurons generate ectopic afferent signals to induce abnormal sensory processing and further bombard spinal or supraspinal structure to result in central sensitization in chronic pain condition. Our study showed that ATF3 expression was markedly and rapidly increased in the contralateral uncompressed DRG neurons from postoperative day 1. It is very likely that primary sensory neurons became hyperexcitable due to peripheral neuronal damage, in turn contributing to the development and maintenance of contralateral hypersensitivity in the mCCD rat. Injury signals might be generated and transmitted from injured DRG neurons to the blood stream via neurotrophic factors or directly to the spinal cord where retrograde signals might be generated and transmitted from the spinal cord to the contralateral DRGs. Target-derived trophic factors from injured and degenerated DRG neurons and axons may be acting on DRG neurons in the contralateral ganglion via blood circulation. Potential trophic factors include neurotrophic growth factor and neurotrophin 3 [24].

Increasing evidence demonstrates that glial cell activation may be involved in the development and maintenance of bilateral pain [25, 26]. Although it is reported that ATF3 expression was upregulated in satellite cells of the DRG after an injury to the sciatic nerve [27], it was not observed in the present study. It is likely that the expression of ATF3 in satellite cells is much weaker than that in sensory neurons. Our study showed that CGRP expression was markedly and rapidly increased in the contralateral large- and medium-sized DRG neurons only after the mCCD. CGRP has been regarded as a signaling molecule mediating the interactions between damaged neurons and surrounding glial cells, and it activates both astrocytes and microglia at the transcriptional level [28, 29]. Thus, CGRP might be involved in the process of bilateral pain through the activation of astrocytes and microglia. This activation might be mediated through calcium waves or oscillations spread within astroglial networks that could then promote new synaptic connections in the spinal cord [30]. If so, strong signals from injured DRGs transmitted to the ipsilateral spinal cord may also amplify calcium waves or oscillations of astrocytes that then activate the contralateral spinal cord and finally spread to uncompressed DRGs.

Ectopic spontaneous activity of injured DRG neurons is involved in glial activation and the development of pathological pain [5, 31, 32]. It seems likely that peripheral satellite glial cells might be activated by the spontaneous activity of compressed DRG neurons and that the activation of glial cells might further contribute to bilateral pain following the chronic compression of multiple DRGs. Activated glia release proinflammatory cytokines, such as IL-1, IL-6, and TNF-alpha, which lead to further activation of glia and act as a secondary stimulus [33].

It is still not known whether the signaling of bilateral hypersensitivity operates primarily at the nociceptor, spinal, thalamic, or cerebral cortical levels. One speculation is that the signaling may be mediated via the commissure tracts in the spinal cord, where signaling via interneurons is aided by growth factors such as the brain-derived neurotrophic factor (BDNF) and neurotrophin 3 [34]. Another possibility is that substances may be released from injured sensory neurons which carry a “pain message” to the brain, which promote bilateral descending pain facilitating pathways [35]. Further studies are necessary to investigate the origins of contralateral hypersensitivity in the central and peripheral systems.

## 5. Conclusions

Our results showed a contralateral hypersensitivity by the chronic compression of multiple DRGs and neuropathic changes in contralateral primary sensory neurons that may be involved in the induction and the maintenance of contralateral hypersensitivity including tactile allodynia, thermal hyperalgesia, and cold allodynia. Understanding signaling mechanisms and pathogenesis underlying contralateral sensitization, specifically in the case of chronic low back pain, will have novel therapeutic targets for the management of treatment of pain. Based on our study, one potential treatment site may target at the level of uncompressed contralateral sensory neurons, which might have some success in treating intractable phantom pain.

## Supplementary Material

Supplementary Figure 1: Correlations between evoked pain behaviors and ATF3 expression in the ipsilateral and contralateral DRGs on the postoperative 1st day following mCCD.
Supplementary Figure 2: Correlations between evoked pain behaviors and CGRP expression in the ipsilateral and contralateral DRGs on the postoperative 1st day following mCCD.
