# Supplementary material for: Bilateral Neuropathy of Primary Sensory Neurons by the Chronic Compression of Multiple Unilateral DRGs
Source: Neural Plast. 2015 Dec 27;2016:2130901. doi: 10.1155/2016/2130901 (PMC4706945; doi:10.1155/2016/2130901)
Supplement: Supplementary file 1 — Supplementary Figure 1: Correlations between evoked pain behaviors and ATF3 expression in the ipsilateral and contralateral DRGs on the postoperative 1st day following mCCD. Supplementary Figure 2: Correlations between evoked pain behaviors and CGRP expression in the ipsilateral and contralateral DRGs on the postoperative 1st day following mCCD. [file 2130901.f1.pdf]

Supplementary figure legends

SupplFigure 1 Correlations between evoked pain behaviors and ATF3 expression in the ipsilateral and contralateral DRGs on the postoperative 1st day following mCCD. A, Linear regression analysis revealed that mechanical allodynia was positively correlated with the ATF3 expression in both ipsilateral compressed and contralateral DRG neurons. B, Linear regression analysis revealed that cold allodynia was positively correlated with the ATF3 expression in both ipsilateral compressed and contralateral DRG neurons.

SupplFigure2 Correlations between evoked pain behaviors and CGRP expression in the ipsilateral and contralateral DRGs on the postoperative 1st day following mCCD. Linear regression analysis revealed that there were significant correlations after mCCD between CGRP expression in the ipsilateral and contralateral DRGs with A, mechanical allodynia. B, cold allodynia.

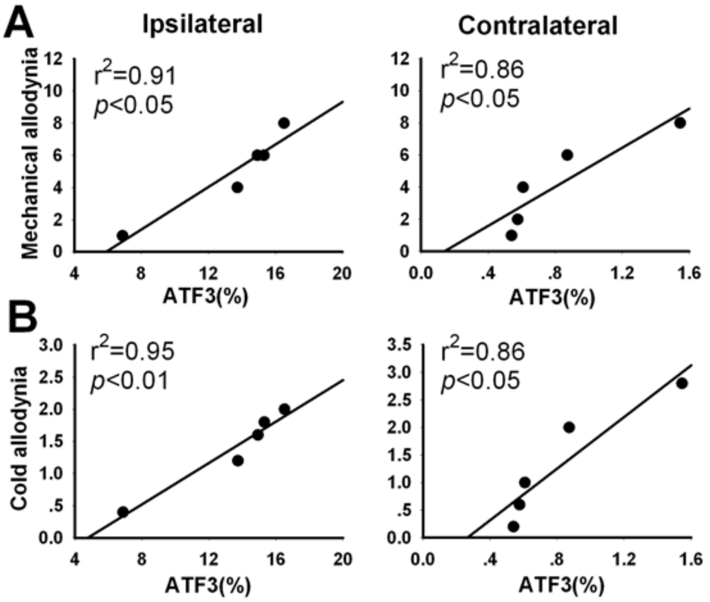

SupplFigure 1

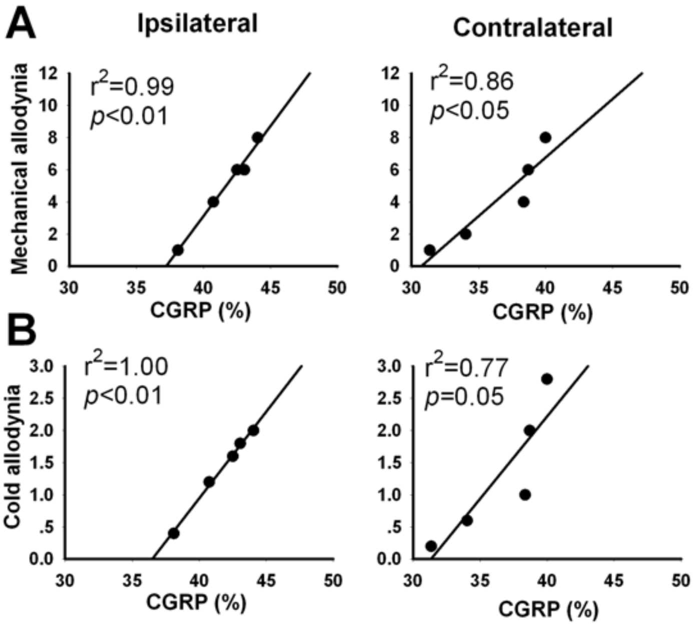

SupplFigure 2
